# Multi-regional radiomics based on planning CT and information complementarity between GTVp and GTVn: an explainable model for predicting 5-year recurrence risk in HPV-positive oropharyngeal cancer

**DOI:** 10.1186/s13014-026-02889-8

**Published:** 2026-07-08

**Authors:** Chunsheng Wang, Mingjun Ding, Xiang Cao, Yizhi Ge, Shanliang Hu, Jianguo Zhang, Linzhi Han, Chenjing Zhu, Xia He

**Affiliations:** 1 Department of Radiation Oncology, Affiliated Cancer Hospital of Nanjing Medical University, & Jiangsu Cancer Hospital & Jiangsu Institute of Cancer Research, Nanjing, Jiangsu 210009 China; 2 Radiotherapy Department, The Affiliated Changzhou NO 2 People’s Hospital of Nanjing Medical University, Changzhou, 213000 China; 3 Nanjing Medical University, Nanjing, Jiangsu 211166 China; 4 Department of Radiation Oncology, Yantai Yuhuangding Hospital, Yantai, Shandong 264000 China; 5 Department of Radiation Oncology, Shandong Provincial Key Laboratory of Radiation Oncology, Shandong Cancer Hospital and Institute, Shandong First Medical University, Shandong Academy of Medical Sciences, Jinan, 250117 China; 6 Department of Radiology, Zhongnan Hospital of Wuhan University, Wuhan, Hubei 430071 China

**Keywords:** Oropharyngeal cancer, Human papillomavirus (HPV), Radiomics, Explainable model, Recurrence

## Abstract

**Purpose:**

This study aims to develop and validate a multi-regional radiomics machine learning model integrating the spatial heterogeneity features of both the primary gross tumor volume (GTVp) and metastatic lymph nodes (GTVn) to explore its potential as a non-invasive tool for estimating the 5-year recurrence risk of HPV-positive oropharyngeal carcinoma (OPC) patients.

**Methods:**

This study included 716 HPV + OPC patients from three independent datasets (training set: 390; internal validation set: 166; independent test set: 160). PyRadiomics was used to extract radiomic features from each target volume. Ten classical machine learning models were constructed, and the SHAP algorithm was applied to quantify feature contributions and optimize core variables. A clinical-radiomics model based solely on GTVp and a clinical-radiomics model based on a feature-level fusion strategy (GTVp&n) were established respectively.

**Results:**

The XGBoost model based on the GTVp&n strategy showed the best performance, with AUCs of 0.907, 0.852, and 0.835 in the training, internal validation, and independent test set, respectively—significantly higher than those of the single GTVp model and the GTVpn (fused target volume) model. A nomogram incorporating clinical factors demonstrated good discrimination, calibration, and clinical utility across all datasets. NRI (0.242) and IDI (0.097) analyses confirmed that this combined model significantly improved risk reclassification capability compared to the GTVp-based clinical-radiomics model.

**Conclusion:**

A multi-regional radiomics model integrating features from both the primary lesion and cervical lymph nodes significantly enhances the predictive performance for recurrence risk in HPV + OPC. This model facilitates individualized recurrence risk stratification and may generate research hypotheses for future exploration of personalized de-escalation or intensified treatment strategies, as well as supporting individualized follow-up management.

**Supplementary Information:**

The online version contains supplementary material available at https://doi.org/10.1186/s13014-026-02889-8.

## Introduction

Oropharyngeal carcinoma (OPC) represents an increasing subtype of head and neck malignancies, with its etiology well established in human papillomavirus (HPV) infection [1, 2]. HPV infection has replaced traditional factors such as smoking and alcohol consumption and become the primary driver of the global rise in OPC incidence [2, 3]. Clinical evidence underscores that HPV-positive (HPV+) OPC possesses a distinct biological profile, characterized by high radioresponsiveness and a superior prognosis, resulting in a significantly higher 5-year survival rate (approximately 70%–80%) than HPV-negative OPC patients [4–6]. However, despite the generally optimistic prognosis, approximately 15%–25% of patients experience local recurrence or distant metastasis after radical chemoradiotherapy, and once recurrence occurs, patients often respond poorly to subsequent salvage treatment [7–9]. The current TNM staging system has certain limitations in evaluating these patients, as it is primarily based on the anatomical extent of the tumor and may not fully encompass the complex biological heterogeneity within the tumor [10, 11]. Therefore, there is a clinical need for predictive models that can identify high-risk individuals for recurrence early and accurately, to optimize individualized de-escalation or intensification treatment decisions.

Radiomics, as a high-throughput feature extraction technique, can transform standard medical images into quantitative spatial features, thereby offering a non-invasive mathematical tool to estimate radiographic spatial heterogeneity [12, 13]. Although some studies have attempted to build predictive models using radiomic features of the primary tumor, the robustness of these models in cross-center validation requires further verification [14, 15]. A key point that has been long overlooked is that HPV + OPC has a strong lymphotropic nature, and its most significant clinical feature is early and massive cervical lymph node metastasis [16]. Metastatic lymph nodes serve as anatomical markers of disease progression, and their imaging phenotypes may act as potential surrogate indicators correlating with tumor invasiveness, immune features and metastatic propensity [16]. Their metastatic status (including lymph node size, number, and extranodal extension) is not only a core indicator of tumor staging but also a key basis for recurrence risk stratification [2, 16, 17].

HPV + OPC patients with cervical lymph node metastasis have a two- to threefold higher risk of recurrence compared to those without metastasis, and the imaging characteristics of the metastatic lymph nodes are closely related to tumor aggressiveness and treatment resistance [2, 18]. Biologically, morphological and textural features within metastatic lymph nodes, such as extranodal extension (ENE), cystic changes, or complex necrosis patterns, often indicate stronger radioresistance and a higher risk of hematogenous metastasis [19]. For instance, although radiological ENE or prominent keratinizing low-attenuation parameters typically portend a dismal survival and dismal local control in HPV-negative squamous cell carcinomas, they fail to demonstrate a similarly aggressive predictive power or direct association with regional recurrence in HPV-positive cohorts [20]. The observed biological discordance poses a challenge to purely anatomy-based risk stratification. It indicates that metastatic lymph nodes may represent an additional biological dimension in HPV-positive OPC, which could be associated with the tumor’s overall phenotypic aggressiveness, beyond their role as secondary anatomical sites [21]. Imaging information extracted solely from the primary gross tumor volume (Gross Tumor Volume of primary lesion, GTVp) cannot fully characterize the complete risk profile of the patient. The primary tumor and cervical metastatic lymph nodes (Gross Tumor Volume of nodal metastasis, GTVn) exhibit significant differences and complementarities in spatial distribution, blood supply, and immune infiltration patterns [22, 23]. Therefore, performing a “multi-regional combined analysis” of the primary tumor and lymph nodes as independent units, by exploring the potential synergistic prognostic weight between them, is expected to capture more profound spatial heterogeneity information, thereby significantly improving the discriminative accuracy and generalization ability of predictive models. To capture these sub-visual biological variances within the localized microenvironment, recent computational oncology has advanced toward multi-regional or habitat-based radiomics pipelines, demonstrating that partitioning the tumor volume into distinct spatial subregions captures phenotypic heterogeneity more effectively than traditional whole-tumor averaged approaches [24]. However, the optimal paradigm to decouple and synergistically fuse independent digital phenotypes derived from both the primary gross tumor volume and the metastatic nodal volume (GTVn) remains to be further elucidated [25]. A critical methodological challenge that has long been overlooked in the conventional paradigm of physically merging all targets (GTVpn) is the vast discrepancy in volume and voxel counts between the primary lesion and metastatic lymph nodes. This disparity inevitably induces a ‘signal dilution’ effect, whereby the dominant signals from the primary tumor easily mask the subtle yet critical textural fingerprints within smaller lymph nodes. To address this limitation, the present study proposes a decoupled multi-regional feature-level combination strategy (GTVp&n), which aims to mitigate this signal dilution bottleneck, thereby quantifying and isolating the independent diagnostic weight and complementary information contributed by cervical nodal textures for 5-year recurrence risk stratification.

This study, based on multi-center real-world data, aims to develop and validate a multi-regional radiomics machine learning model based on planning CT. Through a rigorous feature-level combination strategy, we compared the effectiveness of different modeling schemes, including the primary tumor and fused target volume, and focused on clarifying the added value of lymph node radiomics information in predicting the 5-year recurrence risk of HPV + OPC patients. This study aims to provide scientific evidence for recurrence risk stratification, individualized treatment decisions, and follow-up management of HPV + OPC patients, ultimately improving patient survival outcomes.

## Methods

### Data acquisition and patient cohorts

This study adopts a retrospective multicenter design. Planning CT images and clinical data of patients with OPC were obtained from the public database of The Cancer Imaging Archive (TCIA)(https://www.cancerimagingarchive.net/). The datasets include HNSCC [26], RADCURE [27], and OPC-Radiomics [28]. To eliminate any ambiguity regarding the endpoint formulation, the prediction of 5-year recurrence risk in this investigation was strictly framed as a stationary binary classification task. The prediction label was defined as follows: patients who sustained any tumor recurrence events (including local recurrence, regional failure, or distant metastasis) within the 5-year post-treatment follow-up window were assigned to the recurrence group; conversely, individuals who successfully completed a minimum follow-up duration of 5 years with no evidence of recurrence were assigned to the non-recurrence group. To preclude temporal windows bias, censored data with an insufficient follow-up duration below 5 years without sustaining an event were rigorously excluded from model development. Inclusion criteria were strictly limited to: age ≥ 18 years; histopathologically confirmed oropharyngeal squamous cell carcinoma with clearly labeled primary anatomical sub-sites, including the base of tongue, tonsils, soft palate, lateral pharyngeal wall, and posterior pharyngeal wall; HPV status determined as positive, defined as diffuse and strong positive p16 protein immunohistochemical staining (positive cells ≥ 70%); received radiotherapy or concurrent chemoradiotherapy with complete radiotherapy target volume contours, including GTVp and GTVn; radiotherapy regimens must clearly record the total dose, fractionation, and whether combined with chemotherapy; clinical follow-up time ≥ 5 years, or follow-up until a tumor recurrence event occurred. The follow-up endpoint was defined as the first occurrence of tumor recurrence, including local recurrence, regional recurrence, and distant metastasis, with recurrence confirmed by imaging or clinical follow-up. Exclusion criteria were: concurrent primary malignant tumors at other sites; CT images with severe artifacts, including metal implant artifacts, respiratory motion artifacts, or artifacts caused by scanner malfunction, affecting the accuracy of target contouring and feature extraction; missing key clinicopathological information, including age, sex, tumor stage, treatment regimen, or follow-up outcome. Due to the intrinsic constraints of legacy multicenter public repositories (TCIA), digital radiotherapy dose distribution files were largely unavailable or cross-centrally incompatible across the historical cohorts. Consequently, dosiometric parameters or dosiomic features were not introduced in the current modeling phase. This study specifically positions itself as a pre-treatment quantitative tool utilizing baseline anatomical Planning CT scans to decipher spatial heterogeneity. To address the inherent heterogeneity across the multicenter public domain datasets, detailed image acquisition parameters-including slice thickness, pixel spacing, tube voltage (kVp), exposure (mAs), manufacturer, and convolution kernels-were systematically collected and summarized in Supplementary Table 2. Patients from the RADCURE and OPC-Radiomics datasets were merged into one dataset and randomly divided into a training set and an internal validation set at a 7:3 ratio, with the HNSCC dataset serving as an independent test set. The study protocol was reviewed and approved by the institutional medical ethics committee. Since the data are from public open resources, the process of obtaining written informed consent was waived.

### Lesion segmentation and consistency verification

Radiotherapy planning CT images and original contour files for all included patients were imported into the open-source medical image processing software 3D Slicer (version 4.11.0) for target standardization and consistency verification. Regions of interest (ROI) were independently contoured by two radiation oncologists with over 10 years of experience. The contours for GTVp and GTVn were based on confirmed outlines in the radiotherapy planning system and merged into a fused target volume. To evaluate inter-observer consistency, 30% of cases were randomly selected via simple random sampling to calculate the Dice Similarity Coefficient and the 95th percentile Hausdorff Distance. An average Dice coefficient > 0.85 and an average Hausdorff distance < 2 mm were considered good consistency. All contouring was performed while blinded to clinical outcomes to avoid bias.

### Radiomic feature extraction and preprocessing

Image preprocessing included the following steps: (1) voxel resampling to isotropic 1 × 1 × 1 mm³ using B-spline interpolation to preserve texture information; (2) gray-level histogram Z-score normalization using adipose tissue at the same level (ROI placed in subcutaneous fat at the C3 vertebral level) as a reference. Feature extraction was performed using the PyRadiomics plugin (version 3.0) in 3D Slicer. A total of 851 features were extracted, covering the following categories: (1) morphological features (*n* = 14): e.g., volume, surface area, sphericity; (2) first-order statistical features (*n* = 18): e.g., entropy, skewness, and kurtosis of the intensity histogram; (3) texture features (*n* = 75): including Gray Level Co-occurrence Matrix (GLCM), Gray Level Run Length Matrix (GLRLM), and Neighborhood Gray Tone Difference Matrix (NGTDM); (4) wavelet features (*n* = 744): higher-order textures from 8 sub-bands based on Haar wavelet decomposition. Feature stability was evaluated via two sets of contours from 30 patients, and features with an Intra-class Correlation Coefficient (ICC) > 0.8 were retained for subsequent analysis. We removed features with ICC < 0.8 to eliminate scanner-related batch effects following IBSI radiomics standardization guidelines.

### Feature dimension reduction and selection

Feature reduction was performed in four steps: (1) elimination of low-stability features with ICC < 0.8; (2) elimination of statistically insignificant features via univariate analysis (Mann-Whitney U test, *P* < 0.05); (3) calculation of Pearson Correlation Coefficients (PCC) to exclude highly redundant features (|r| > 0.85); (4) further dimension reduction using the Least Absolute Shrinkage and Selection Operator (LASSO) regression model (with 10-fold cross-validation for penalty parameter selection) on the optimized feature set to screen radiomic features closely related to 5-year recurrence in HPV + OPC patients. The selected features were used directly for subsequent model construction.

### Machine learning model construction and optimization

Ten classic machine learning algorithms, including Logistic Regression, Random Forest, SVM, LightGBM, XGBoost, and GBDT, were trained using LASSO-selected features to predict 5-year recurrence risk. Full model definitions and abbreviation lists have been condensed for brevity. Hyperparameters were optimized using grid search, with AUC from 5-fold cross-validation as the evaluation metric. The best-performing model was analyzed using the SHapley Additive exPlanations (SHAP) algorithm to quantify feature importance and calculate the contribution of each feature to the model’s predictions. SHAP summary plots, dependence plots, and force plots were generated to visualize the direction and strength of the association between features and recurrence risk. The machine learning model was retrained using the final core features, and the radiomics score (R-score) for each patient was calculated based on the predicted probability of the optimal model, with higher R-scores indicating higher 5-year recurrence risk. All model training was repeated 100 times using bootstrap to calculate confidence intervals for performance metrics.

### Construction and validation of combined predictive model

Clinical factors were screened for independent predictors via univariate and multivariate logistic regression analyses. A nomogram was constructed by combining independent prognostic factors with the R-score predicted probability from the machine learning model. Model performance was validated in the training set, internal validation set, and independent test set. Validation metrics included discrimination, calibration, and clinical utility. Discrimination was assessed by calculating AUC values via ROC curve analysis, and the improvement in discrimination of the combined nomogram compared to the radiomics-only model (R-score only) was compared using the Net Reclassification Improvement (NRI) and Integrated Discrimination Improvement (IDI). Calibration curves (Bootstrap = 1000 times) were used to test the consistency between predicted probabilities and actual recurrence rates. Decision Curve Analysis (DCA) and clinical impact curves evaluated clinical utility.

### Multi-regional model comparison and interpretability analysis

In the subgroup of patients with lymph node metastasis, radiomics models were constructed based on GTVp, GTVpn (fused target volume), and GTVp&n (combined features). The AUC values of the three models were compared using the DeLong test. After selecting the optimal model, the nomogram construction process described in Step 6 was repeated. Model performance was validated in the training and internal validation sets using AUC, NRI, IDI, calibration curves, and DCA to comprehensively compare the predictive performance between the new combined nomogram model and the GTVp-based combined nomogram model, and to evaluate the added value of including lymph node target volume features. SHAP was used to analyze the interpretability of the new combined nomogram model and to clarify the contribution direction and degree of core radiomics features and clinical factors to the predictions.

### Statistical analysis

Categorical variables are described as counts and percentages, with inter-group comparisons performed using the χ² test or Fisher’s exact test (as appropriate). Continuous variables are described as mean ± standard deviation if they follow a normal distribution, with comparisons performed using independent samples t-tests; otherwise, they are described as median (interquartile range, IQR), with comparisons using the Mann-Whitney U test. Multiple comparisons were corrected using the Bonferroni method. All statistical tests were two-sided, and *P* < 0.05 was considered statistically significant. Statistical analysis was performed using R software (version 4.2.0) and Python (version 3.8).

## Results

### Research design and analysis workflow

**Fig. 1:**
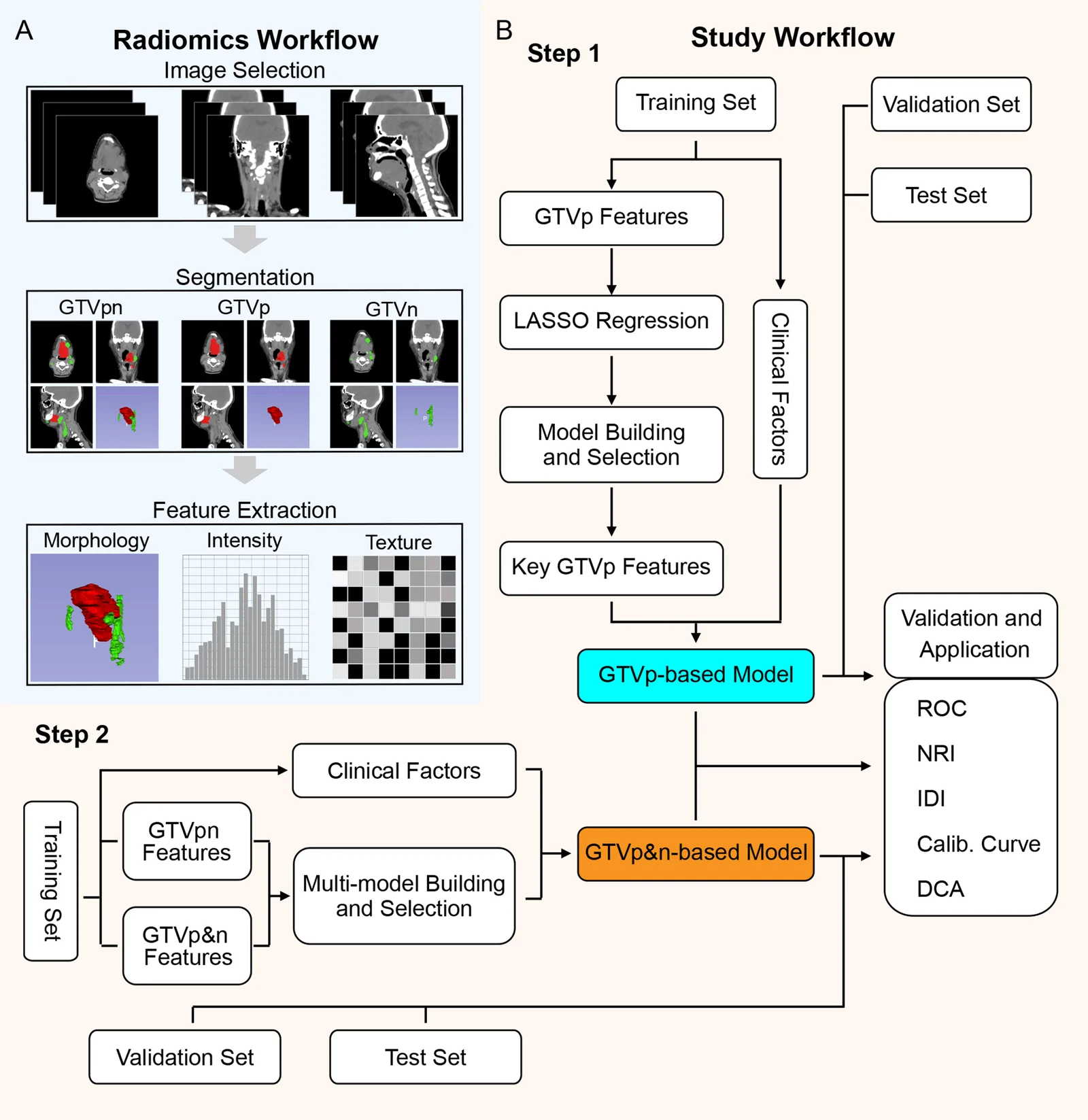
Overall study workflow and radiomics analysis pipeline

The overall design and analysis workflow of this study is illustrated in Fig. 1, covering the complete stages of multicenter dataset acquisition, radiotherapy simulation CT image preprocessing, target volume segmentation and consistency verification, radiomic feature extraction and screening, construction and optimization of multiple types of machine learning models, prognostic model construction based on different target volumes (GTVp, GTVpn, GTVp&n), internal and independent test, as well as inter-model performance comparison and interpretability analysis, ensuring a closed logic loop and reliability of results. The workflow is divided into two core modules: first, the radiomics workflow, including image screening, target segmentation, feature extraction (morphology, intensity, and texture features), LASSO regression feature selection, and determination of key features; second, the research workflow, including clinical factor collection and analysis, multi-model construction and selection, validation and application of the GTVp-based baseline model, as well as the construction, performance comparison, and validation via calibration curves and DCA of multi-regional models, ultimately achieving a comprehensive evaluation of the models in the training, validation, and testing sets.

### Patient baseline characteristics

A total of 716 eligible HPV-positive oropharyngeal cancer patients were included in this study, including 390 in the training set, 166 in the internal validation set, and 160 in the independent test set. The baseline clinicopathological characteristics of the three groups are shown in Table 1, covering key indicators such as age, sex, smoking history, tumor T stage, N stage, clinical stage, and whether combined with chemotherapy. Statistical analysis showed that there were no statistically significant differences in the distribution of baseline characteristics between the training set and the independent test set (all *P* > 0.05). Specifically, the proportions of patients aged ≥ 60 years were 55.4% and 52.4%, respectively; male patients accounted for 83.6% and 83.7%; patients with a positive smoking history accounted for 66.4% and 65.7%; patients with T3–4 stage tumors accounted for 49.0% and 42.2%; patients with clinical stage IV accounted for 13.1% and 13.3%; and patients receiving combined chemotherapy accounted for 56.2% and 58.4%. Statistical analysis showed no significant differences in all baseline variables between the training set and internal validation set (all *P* > 0.05). After additional statistical comparison between the independent test set and pooled cohort combining training and internal validation data, significant intergroup disparities existed in age, T classification, overall clinical stage and chemotherapy status (all *P* < 0.001) accompanied by elevated SMD values, whereas gender and N classification kept comparable distribution without statistical difference (*P* > 0.05, SMD < 0.2). The detailed constituent ratios of each variable in three cohorts were listed in Table 1. Inherent baseline imbalance of partial indicators derived from multicenter public datasets could not be completely eliminated.

**Table 1:** Baseline characteristics of patients

Variables	Training set *n* = 390	Validation set *n* = 166	*P* ^a^	Test set *n* = 160	*P* ^b^	SMD ^c^
Age			0.092		< 0.001	0.355
≥ 60	216(55.4%)	87(52.4%)		58(36.3%)		
< 60	174(44.6%)	79(47.6%)		102(63.7%)		
Gender			0.966		0.628	0.055
Male	326(83.6%)	139(83.7%)		137(85.6%)		
Female	64(16.4%)	27 (16.3%)		23(14.4%)		
Smoking			0.865		0.054	0.181
Yes	259(66.4%)	109(65.7%)		92(57.5%)		
No	131(33.6%)	57(34.3%)		68(42.5%)		
T			0.141		< 0.001	0.380
1–2	199(51.0%)	96(57.8%)		111(69.4%)		
3–4	191(49.0%)	70(42.2%)		49(30.6%)		
N			0.569		0.993	0.012
0	34(8.7%)	17(10.2%)		14(8.8%)		
1–3	356(91.3%)	149(89.8%)		146(91.2%)		
Stage			0.949		< 0.001	1.458
I-II	48(12.3%)	22(13.3%)		14(8.8%)		
III	291(74.6%)	122(73.4%)		23(14.4%)		
IV	51(13.1%)	22(13.3%)		123(76.8%)		
Chemotherapy			0.619		< 0.001	0.672
Yes	219(56.2%)	97(58.4%)		136(85.0%)		
No	171(43.8%)	69(41.6%)		24(15.0%)		

a: Comparison of clinical characteristics between training and validation sets. b: Comparison of clinical characteristics between test set and training + validation sets. c: Between test set and training + validation set

For the subgroup of patients with cervical lymph node metastasis, their baseline characteristics are shown in Supplementary Table 1. This subgroup included 637 patients (352 in the training set, 152 in the test set, and 133 in the validation set). Similarly, no statistically significant differences were found in the distribution of baseline characteristics among these groups (all *P* > 0.05), providing a balanced sample base for subsequent construction and comparison of multi-regional models.

### Construction of the GTVp-based radiomics model

**Fig. 2:**
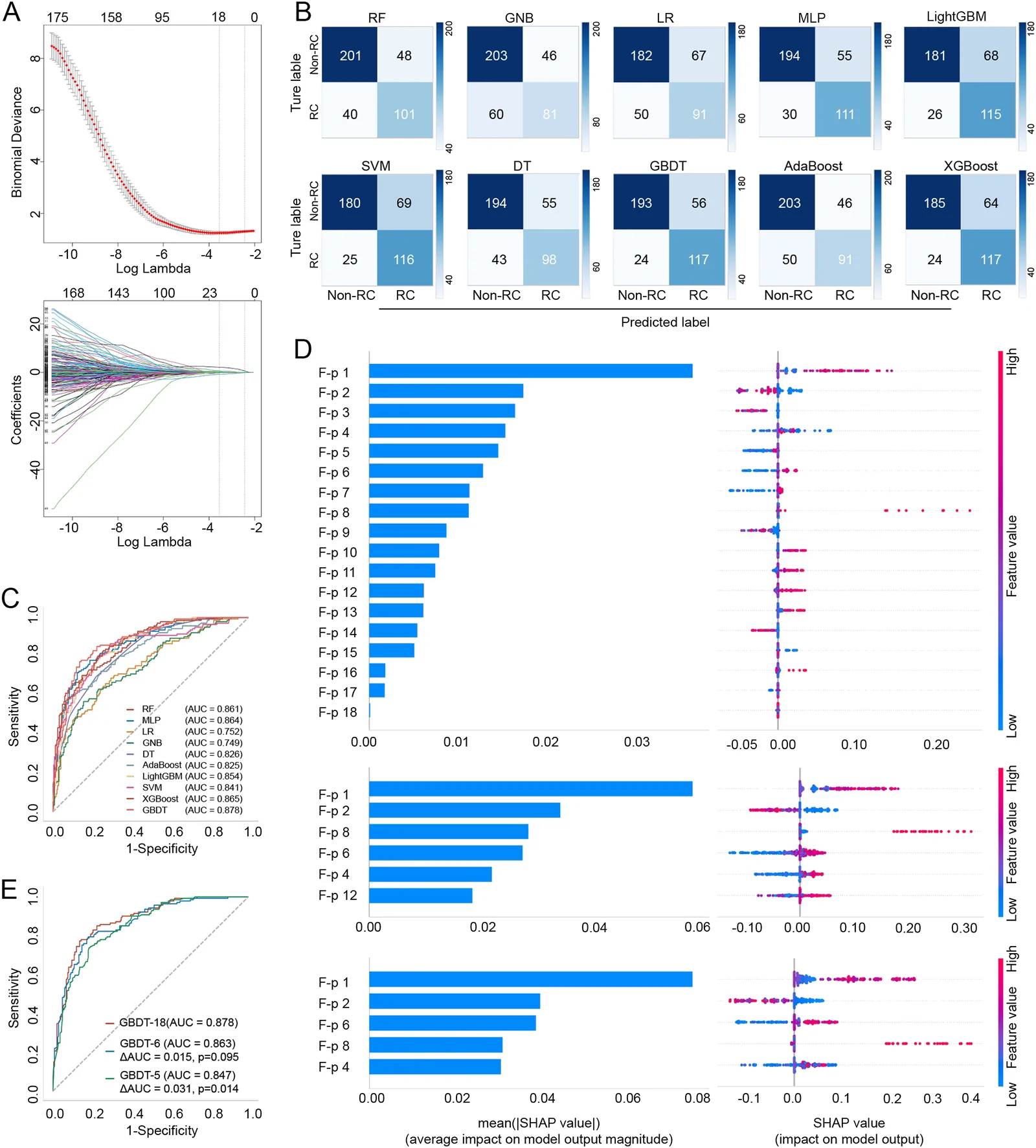
Radiomics feature selection and model performance analysis based on GTVp. (**A**). LASSO logistic regression feature selection curves: the left plot shows the trend of binomial deviance as a function of the logarithm of the penalty parameter (Log Lambda), while the right plot shows the trajectories of feature coefficients relative to Log Lambda; the dashed line indicates the selection threshold corresponding to the optimal penalty parameter. (**B**). Confusion matrices for 10 machine learning models (RF, GNB, LR, MLP, LightGBM, SVM, DT, GBDT, AdaBoost, XGBoost), displaying the number of matches between actual and predicted labels for the non-recurrence (Non-RC) and recurrence (RC) groups. (**C**). ROC curves and corresponding AUC values for the 10 models. (**D**). SHAP summary plot: the horizontal axis represents the SHAP value, the vertical axis represents 18 candidate features selected by LASSO (F-p 1 ~ F-p 18), and color intensity indicates the feature value magnitude (red for high, blue for low). (**E**). Comparison of ROC curves for the GBDT model with different feature counts (18, 6, 5), denoted as GBDT-18, GBDT-6, and GBDT-5

A total of 851 radiomic features were extracted from the GTVp region. After stability analysis and univariate screening, LASSO regression was used for further dimension reduction (Fig. 2A), and 18 features significantly associated with 5-year recurrence were ultimately selected (Supplementary Table 3), including morphological features (e.g., Flatness, Surface Area), first-order statistical features (e.g., Interquartile Range, Kurtosis), and texture features (e.g., Gray Level Non-Uniformity, Autocorrelation). Based on these 18 features, 10 machine learning models were constructed in the training set. The confusion matrices for each model are shown in Fig. 2B, intuitively displaying the predictive classification results for the recurrence group (RC) and non-recurrence group (Non-RC). The comparison of ROC curves for the 10 models is shown in Fig. 2C, where the GBDT model’s ROC curve is at the top, suggesting the best discriminative ability. The predictive performance of each model is shown in Supplementary Table 4, among which the GBDT model performed best with an AUC of 0.878 (95% CI: 0.842–0.913), accuracy of 0.831, sensitivity of 0.773, and specificity of 0.863; followed by the XGBoost model (AUC = 0.865, 95% CI: 0.835–0.891) and the MLP model (AUC = 0.864, 95% CI: 0.832–0.894), while the GNB model performed relatively poorly (AUC = 0.749, 95% CI: 0.697–0.805). Feature importance analysis using the SHAP algorithm (Fig. 2D) revealed no significant difference in model performance when including 6 features compared to 18 features (*P* > 0.05) (Fig. 2E); further reducing the number of features led to a significant decrease in AUC (*P* < 0.05). Therefore, 6 core features were finally determined to construct the GBDT model and obtain the R-score.

### Construction and validation of the GTVp-based combined predictive model

**Fig. 3:**
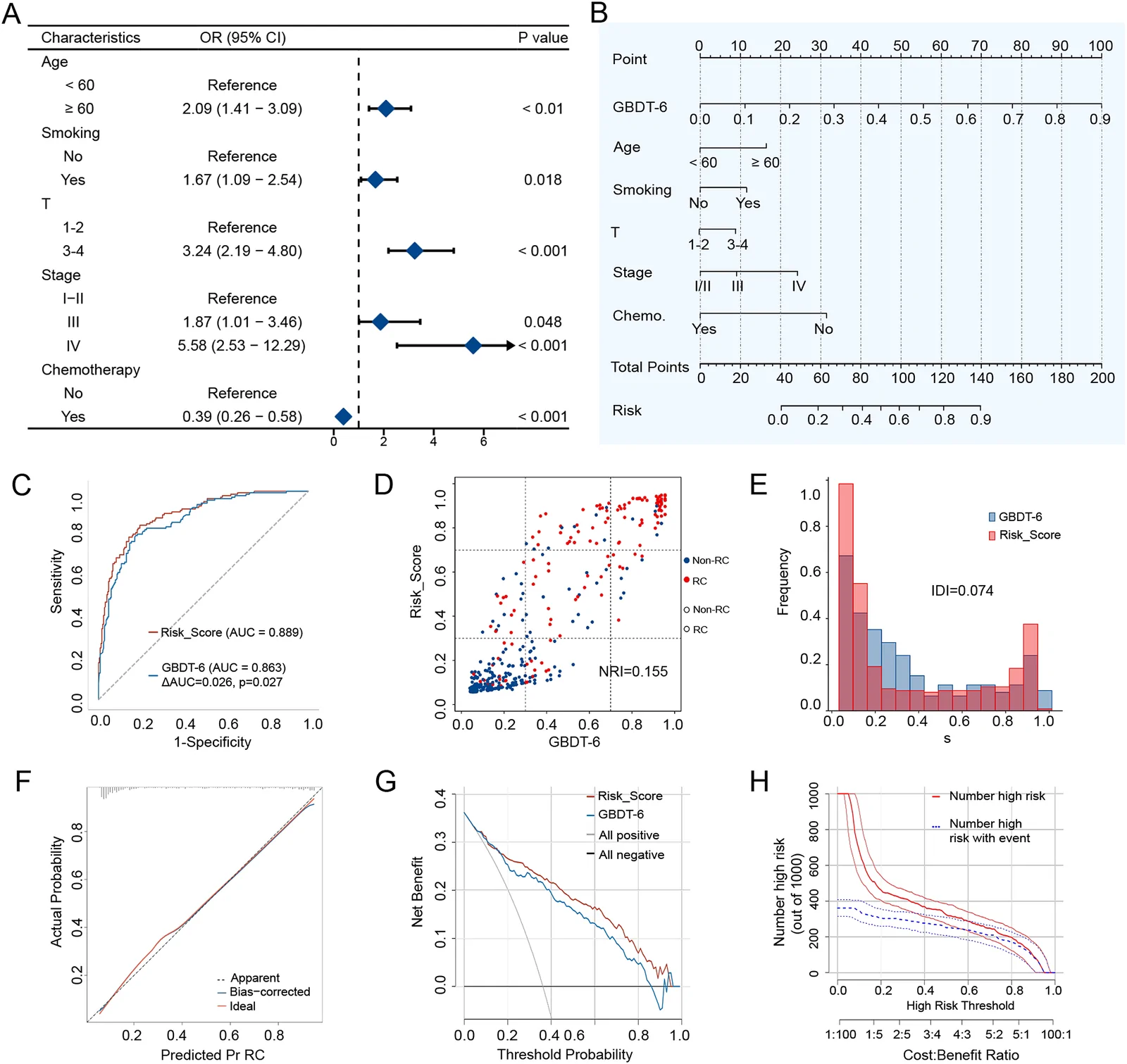
Construction and performance evaluation of the clinical-radiomics combined model based on GTVp. (**A**). Forest plot of the association between clinical factors and 5-year recurrence risk, showing the Odds Ratio(OR), 95% confidence interval (CI), and $P$ value for each variable. (**B**). Nomogram for predicting 5-year recurrence risk, constructed by integrating independent clinical factors and the radiomics score (R-score). (**C**). Comparison of ROC curves between the combined model (Risk_Score) and the single radiomics score (GBDT-6) in the training set. (**D**). Net Reclassification Index (NRI) scatter plot, quantifying the improvement in patient risk stratification provided by the combined model (Risk_Score) relative to the single radiomics score (R-score). (**E**). Integrated Discrimination Improvement (IDI) distribution plot, quantifying the improvement in overall discrimination performance of the combined model relative to the single radiomics score. (**F**). Calibration curve of the combined model for predicting 5-year recurrence probability. (**G**). Decision Curve Analysis (DCA) for the combined model and the single radiomics score, showing the clinical net benefit across different threshold probabilities. (**H**). Clinical impact curve of the combined model, illustrating the correspondence between the predicted number of high-risk cases and the actual number of events at various risk thresholds

Multivariate logistic regression analysis identified age ≥ 60 years (OR 2.09, 95% CI: 1.41–3.09, *P* < 0.01), smoking history (OR 1.67, 95% CI: 1.09–2.54, *P* = 0.018), T stage 3–4 (OR 3.24, 95% CI: 2.19–4.80, *P* < 0.001), clinical stage III (OR 1.87, 95% CI: 1.01–3.46, *P* = 0.048), and clinical stage IV (OR 5.58, 95% CI: 2.53–12.29, *P* < 0.001) as independent risk factors, while chemotherapy was a protective factor (OR 0.39, 95% CI: 0.26–0.58, *P* < 0.001) (Fig. 3A). These clinical factors were combined with the R-score of the above GBDT model to construct a clinical-radiomics combined nomogram model (Fig. 3B). In the training set, the AUC of the combined model was 0.889 (95% CI: 0.856–0.922), significantly higher than that of the radiomics-only model (AUC 0.863, ΔAUC = 0.026, *P* = 0.027) (Fig. 3C, Supplementary Table 5). NRI and IDI analyses showed that the combined model significantly improved reclassification ability compared to the radiomics-only model (NRI 0.15, *P* < 0.01; IDI 0.08, *P* < 0.01) (Fig. 3D, E). Calibration curves showed high consistency between predicted probabilities and actual recurrence rates (Fig. 3F), and DCA indicated higher clinical net benefit for the combined model within the threshold probability range of 0.2–0.8 (Fig. 3G). The clinical impact curve further verified its utility (Fig. 3H).

**Fig. 4:**
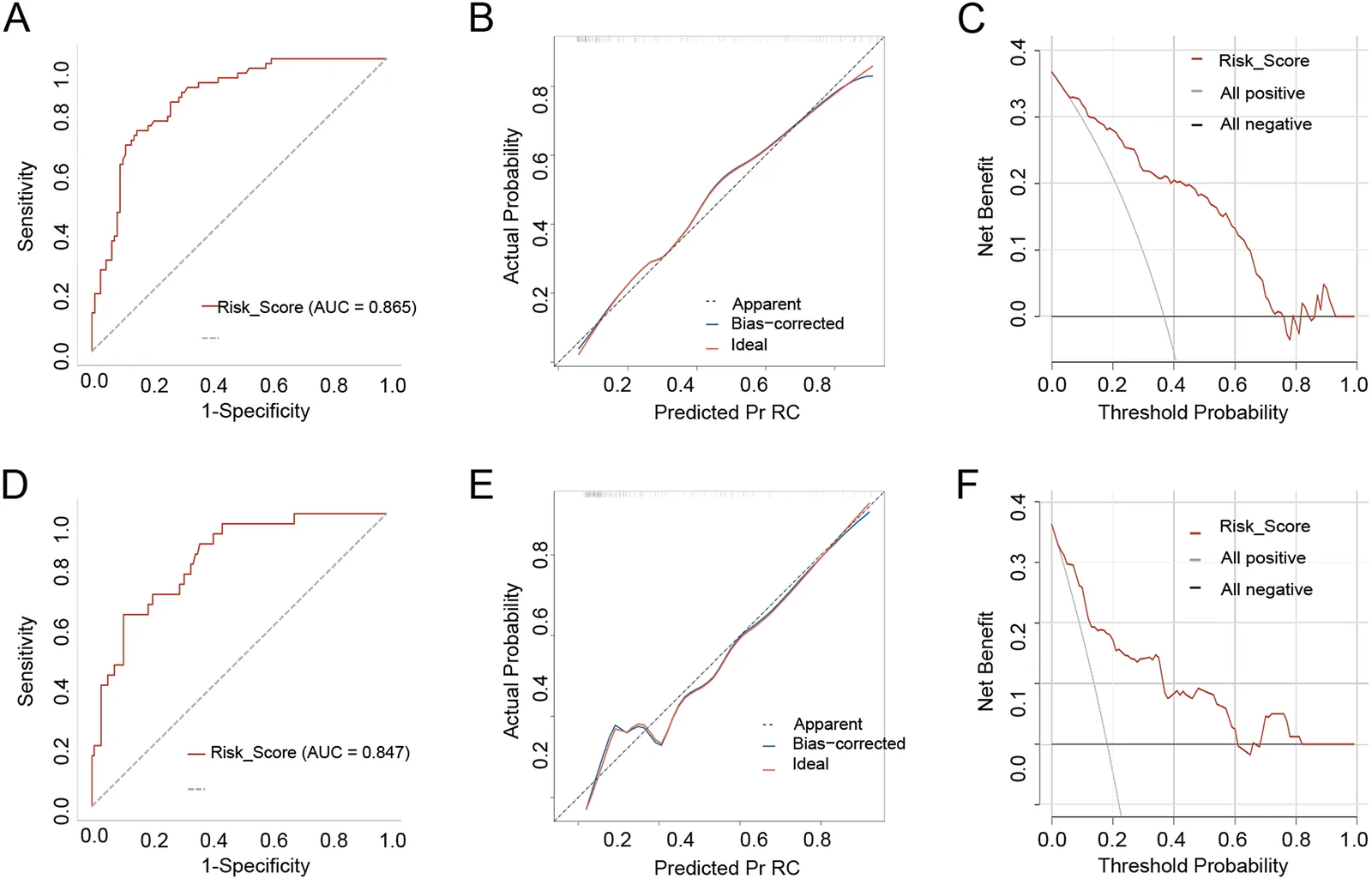
Performance validation of the clinical-radiomics combined model in the internal validation set and external test set. (**A**). ROC curve and corresponding AUC value (0.865) for the combined model (Risk_Score) in the internal validation set. (**B**). Calibration curve of the combined model in the internal validation set, demonstrating consistency between the predicted 5-year recurrence probability and the actual recurrence rate. (**C**). Decision Curve Analysis (DCA) of the combined model in the internal validation set, showing the clinical net benefit across different threshold probabilities. (**D**). ROC curve and corresponding AUC value (0.847) for the combined model (Risk_Score) in the independent external test set. (**E**). Calibration curve of the combined model for predicting 5-year recurrence probability in the independent external test set. (**F**). Decision Curve Analysis (DCA) of the combined model in the independent external test set

In the internal validation set and independent test set, the combined model maintained robust performance. The internal validation set had an AUC of 0.865 (95% CI: 0.810–0.920), accuracy of 0.813, sensitivity of 0.754, and specificity of 0.848; the independent test set had an AUC of 0.847 (95% CI: 0.774–0.919), accuracy of 0.850, sensitivity of 0.655, and specificity of 0.893 (Supplementary Table 5). Figure 4A and D show the ROC curves for the validation and test sets, respectively; calibration curves showed good consistency (Fig. 4B, E), and DCA confirmed clinical applicability across a wide range of thresholds (Fig. 4C, F), indicating good generalization ability.

### Multi-regional radiomics model construction and comparison

**Fig. 5:**
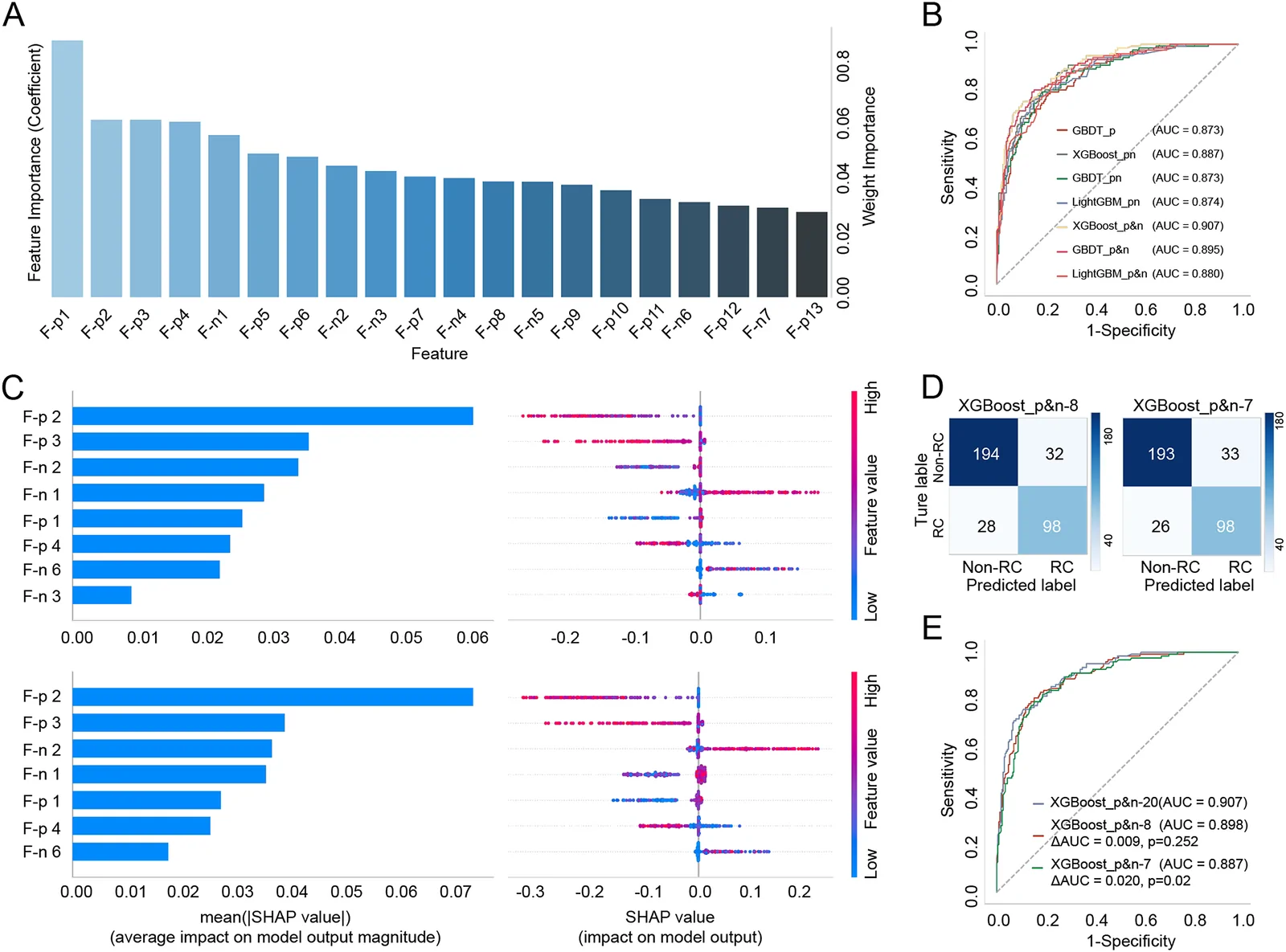
Radiomics model selection and performance comparison based on the multi-regional feature-level fusion (GTVp&n) strategy. (**A**). Feature importance bar chart, showing the distribution of weight coefficients for 20 selected radiomics features. (**B**). Comparison of ROC curves for 7 machine learning models under three strategies: single primary tumor (p), physical fusion (pn), and feature-level fusion (p&n). (**C**). SHAP-guided core radiomics feature selection: the horizontal axis represents SHAP values (reflecting the direction and intensity of a feature’s influence on model output), the vertical axis represents core features, and color intensity indicates the feature value level (red for high, blue for low), showing the distribution of feature values and their influence direction on model prediction. (**D**). Confusion matrices of the two feature-subset models optimized via the SHAP algorithm (XGBoost_p&n-8 and XGBoost_p&n-7) in the training set. (**E**). ROC curve comparison for the XGBoost_p&n model with different feature counts (20, 8, 7)

In the lymph node metastasis subgroup, radiomics models based on GTVpn (fused target volume) and GTVp&n (combined features) were constructed. For the GTVpn model, 20 features were selected via LASSO (Supplementary Fig. 1A, Supplementary Table 6), and for the GTVp&n model, 20 features were selected (including features from both GTVp and GTVn regions) (Fig. 5A, Supplementary Fig. 1B, C, Supplementary Table 7). Ten machine learning models were constructed based on GTVpn and GTVp&n, respectively. The performance of each model in the training set is shown in Supplementary Fig. 1D and E, among which the GTVp&n-based XGBoost model performed best with an AUC of 0.907 (95% CI: 0.877–0.938), accuracy of 0.844, sensitivity of 0.762, and specificity of 0.889; followed by the GTVp&n-based GBDT model (AUC = 0.895, 95% CI: 0.861–0.929) (Supplementary Table 8). The comparison of ROC curves for the three models is shown in Fig. 5B, with the GTVp&n-based XGBoost model showing the best performance(Supplementary Tables 8–9). Through SHAP-guided feature selection (Supplementary Fig. 1F, Fig. 5C-D), 7 key features were ultimately identified as core variables for the GTVp&n-based XGBoost model, resulting in excellent performance and a streamlined structure (Fig. 5E).

### Validation and interpretability analysis of the GTVp&n-based combined predictive model

**Fig. 6:**
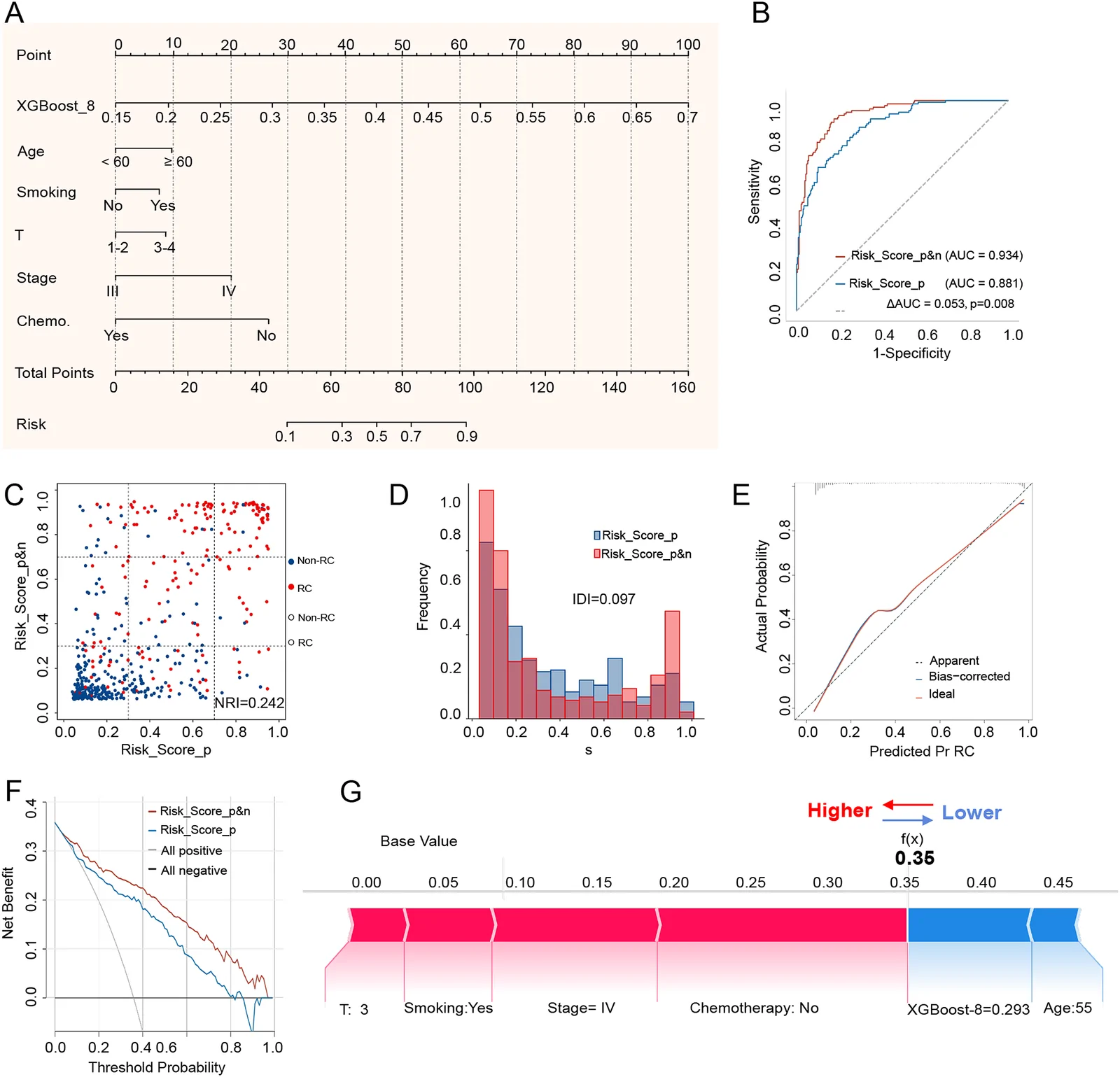
Construction, evaluation, and individual-case interpretability analysis of the clinical-radiomics combined model based on multi-regional features (GTVp&n). (**A**). Multi-regional combined nomogram for predicting 5-year recurrence risk, constructed by integrating the multi-regional radiomics score (XGBoost-8) and independent clinical factors. (**B**). ROC curve comparison between the GTVp&n-based multi-regional combined model (Risk_Score_p&n) and the GTVp-based single-regional combined model (Risk_Score_p) in the training set. (**C**). Net Reclassification Index (NRI) scatter plot, quantifying the improvement in patient risk stratification by the GTVp&n-based multi-regional combined model compared to the GTVp-based single-regional combined model. (**D**). Integrated Discrimination Improvement (IDI) distribution plot, quantifying the enhancement in overall discrimination performance of the multi-regional combined model relative to the single-regional combined model. (**E**). Calibration curve of the multi-regional combined model for predicting 5-year recurrence probability. (**F**). Decision Curve Analysis (DCA) for the multi-regional combined model and the single-regional combined model. (**G**). Individual prediction explanation plot (Force Plot) based on the SHAP algorithm, illustrating the direction and magnitude of contribution from radiomics features and clinical factors for a specific patient toward the final predicted recurrence risk

**Fig. 7:**
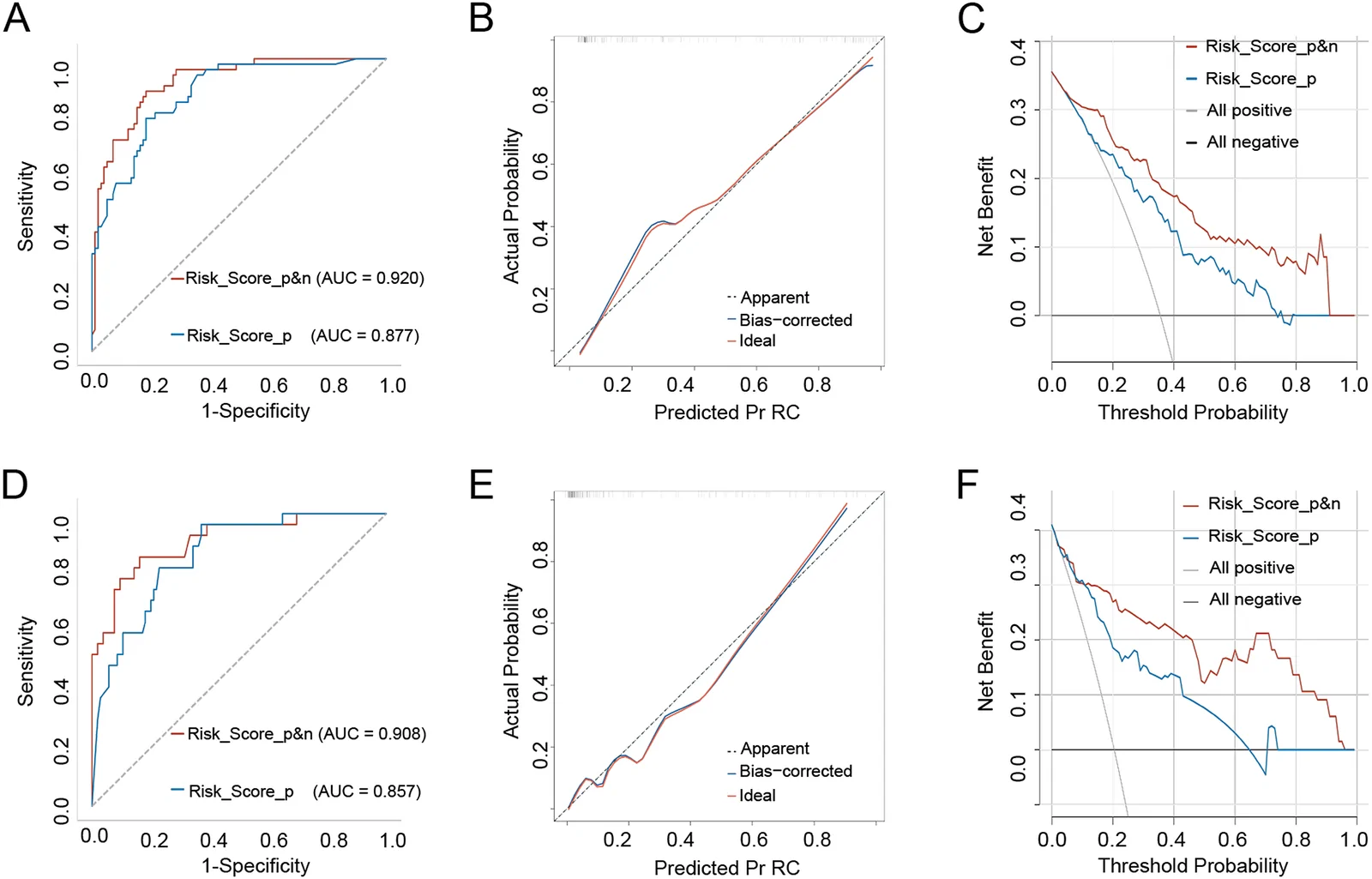
Validation results of the clinical-radiomics combined model based on GTVp&n in the internal validation set and independent test set. (**A**). Comparison of ROC curves between the multi-regional combined model and the single-regional combined model in the internal validation set. (**B**). Calibration curve for the internal validation set, utilizing 1000 bootstrap iterations to show consistency between the predicted 5-year recurrence probability and the actual recurrence rate. (**C**). Decision Curve Analysis (DCA) for the internal validation set. (**D**). Comparison of ROC curves between the multi-regional combined model and the single-regional combined model in the independent external test set. (**E**). Calibration curve for the independent test set. (**F**). Decision Curve Analysis (DCA) for the independent test set

The GTVp&n-based XGBoost radiomics score was combined with independent clinical prognostic factors to construct a new combined nomogram (Fig. 6A). In the training set, the AUC of the new model reached 0.934 (95% CI: 0.908–0.959), a significant improvement over the GTVp-based combined model (AUC 0.881) (ΔAUC = 0.053, *P* = 0.008) (Fig. 6B, Supplementary Table 10). NRI and IDI analyses confirmed improved reclassification (NRI = 0.242, *P* < 0.01; IDI = 0.097, *P* < 0.01) (Fig. 6C, D). Calibration curves showed excellent consistency (Fig. 6E), and DCA indicated higher net benefit in the threshold probability range of 0.1–0.9 (Fig. 6F). SHAP demonstrated the interpretability of the model’s output for specific samples, clarifying the contribution and direction of each feature to the predictive results (Fig. 6G). The AUC of the new combined model in the internal validation set was 0.920 (95% CI: 0.877–0.963), significantly higher than the original model’s 0.877 (*P* = 0.021) (Fig. 7A). Calibration curves and DCA both confirmed its superior performance (Fig. 7B and C). Notably, despite the severe baseline imbalances observed in the independent external test set (which contained a substantially higher proportion of clinical stage IV tumors, younger patients, and intensive chemotherapy regimens compared to the development datasets), our multi-regional GTVp&n combined model maintained an outstanding discriminative capability (AUC = 0.908, 95% CI: 0.842–0.973), which was also significantly higher than that of the original model (0.857, *P* = 0.034), and the calibration and clinical utility remained stable (Fig. 7D and E, and 7F), indicating that the new model had better generalization ability.

## Discussion

Through an in-depth analysis of a multicenter cohort, this study constructed and validated a radiomics model integrating the spatial heterogeneity features of GTVp and GTVn to precisely assess the recurrence risk of patients with HPV + OPC. The research results indicate that the model built on a feature-level combination strategy offers promising prognostic discriminative potential in cross-center validation, with its performance significantly outperforming traditional single-lesion modeling or simple anatomical fusion modeling schemes. This finding provides a non-invasive quantitative tool for the refined stratification of HPV + OPC at the clinical application level, indicating that CT texture features derived from metastatic lymph nodes may carry independent prognostic value statistically associated with recurrence risk.

The core contribution of this study lies in clarifying the importance of cervical metastatic lymph nodes as an independent source of prognostic information. Previous head and neck cancer radiomics endeavors have predominantly relied on whole-tumor features derived strictly from the primary mass to predict locoregional recurrence or molecular status, such as the CT radiomics models developed by Wu et al. (which achieved an AUC of 0.770 in the test set using a combined OPC/HPC advanced cohort) [29] and the whole-tumor classifiers summarized by Stevens et al. [30]. Although recent advanced computational frameworks, such as the unsupervised habitat pipeline proposed by Altinok et al., successfully utilized primary tumor habitats to predict HPV status with high accuracy [31], these conventional approaches systematically omitted the concurrent textural patterns within the metastatic nodal gross volume [15]. HPV + OPC possesses significant lymphophilia, and its complex biological behavior cannot be fully reflected by the primary lesion alone [32]. Metastatic lymph nodes are more than simple secondary anatomical lesions, and their radiomic signatures may supply supplementary prognostic information when combined with primary tumor-derived features [20]. The textural heterogeneity captured within metastatic lymph nodes, as quantified by radiomics, may be associated with the underlying biological aggressiveness of the disease [33, 34]. This study found that the performance improvement of the GTVp&n model compared to the single GTVp model essentially stems from its capture of the information complementarity between the two target volumes during the process of tumor evolution. Imaging features of the primary lesion typically reflect the gene mutation load and metabolic levels during early tumorigenesis [35], While radiomic features of the primary lesion typically capture macroscopic variations during early tumorigenesis, the textural alterations quantified within metastatic lymph nodes may be associated with cross-sectional architectural patterns established during lymphatic dissemination. In the context of radiobiology, statistical heterogeneity parameters derived from CT gray-level distributions—such as Gray Level Non-Uniformity—conventionally serve as radiographic indicators of tissue density variances, which are frequently linked to asymmetric tumor necrosis, disordered cell packing, or regional hypoxic microenvironments [31, 36]. The performance of our multi-regional combined model (AUC = 0.908 in the independent test set) compares favorably with prior radiomics studies focused on recurrence prediction in head and neck cancer. The study by Wu et al., which included both OPC and HPC, reported a test AUC of 0.770 for locoregional recurrence prediction using primary tumor radiomics alone [29]. While direct numerical comparison is constrained by differences in cohort composition (HPV-specific vs. mixed) and outcome definition (5-year recurrence vs. locoregional recurrence), the robust performance of our model in a strictly HPV-positive OPC cohort suggests that a targeted, biologically coherent cohort design, coupled with multi-regional feature integration, may enhance prognostic accuracy. Furthermore, our study complements the primary tumor-centric approach by indicating that nodal radiomic features may offer useful, additional statistical data associated with patient prognosis. However, it must be emphasized that these imaging-based phenotypes provide statistical associations with recurrence risk rather than direct evidence of microscopic biological processes. Because this investigation did not incorporate paired histopathology, immunohistochemistry, or spatial transcriptomic profiles, these macroscopic CT textures should be interpreted strictly as non-invasive typographic correlates of tumor aggressiveness rather than a proof of definitive underlying pathways like active angiogenesis or extracellular matrix remodeling.

At the methodological level, by comparing the performance differences between GTVp&n (feature-level combination) and GTVpn (physical spatial merge) modeling strategies, this study reveals the issue of information dilution in omics analysis. During the physical merge process of GTVpn, the significant differences in volume and voxel counts between the primary lesion and lymph nodes result in the extracted global features being easily dominated by the larger-volume primary lesion, thereby generating a “signal dilution” effect that masks subtle but prognostically valuable texture signals within the lymph nodes [37]. In contrast, the GTVp&n strategy treats the two regions as independent feature sources for high-dimensional combination, maximally preserving their respective unique radiological phenotypes and thus more fully capturing the spatial heterogeneity of the tumor. This finding is highly consistent with the trend in medical artificial intelligence emphasizing “sub-regional analysis” and “independent extraction of multi-source information” [38]. For example, the SC-M_R_ framework proposed by Pranjal Vaidya et al. significantly improved prediction accuracy by fusing macroscopic structural and microscopic pathological features, confirming the importance of independently capturing information from different dimensions for characterizing overall tumor risk [39]. At the same time, this strategy is also consistent with the concept of “tumor habitat radiomics”, which emphasizes that tumors contain spatial subregions with different biological behaviors [40]. Our study suggests that the imaging features derived from the primary lesion and metastatic lymph nodes exhibit complementarity. These findings may offer an alternative analytical perspective to interpret intratumoral spatial heterogeneity and provide a candidate modeling scheme for subsequent multi-lesion radiomic investigations. Our study suggests that the imaging features derived from the primary lesion and metastatic lymph nodes exhibit complementarity. This complementary information, when analyzed jointly, provides a more comprehensive radiomic characterization of the tumor, which is strongly associated with recurrence risk. The significant contribution of nodal (GTVn) radiomic features to our combined model underscores the biological and prognostic relevance of the metastatic nodal microenvironment in HPV-positive OPC. This aligns with prior clinical-imaging studies which have identified nodal characteristics, such as extranodal extension (ENE), as adverse prognostic factors in HPV-positive OPC [25, 41]. For example, Fujita et al. reported that ENE was highly prevalent in HPV-positive patients with nodal metastasis, and Dayan et al. developed an AI model to automate ENE detection from CT, linking it to worse survival [41]. Our radiomic approach provides a quantitative, continuous descriptor of nodal phenotype that may capture subtler variations in nodal biology beyond the binary presence/absence of ENE, potentially explaining its additive value to primary tumor features.

To solve the “black box” problem of machine learning models, this study introduced the SHAP algorithm for interpretability analysis, which not only clarified the direction and intensity of each imaging feature’s contribution but also improved the transparency and clinical interpretability of the model. SHAP analysis helps to clarify which radiomic features drive the model’s prediction for a given patient, moving towards interpretable auxiliary decision-making. SHAP analysis showed that several high-frequency wavelet features (such as Wavelet-HHH texture entropy) and morphological features (such as sphericity) derived from lymph nodes have important predictive weights. Among them, elevated GrayLevelNonUniformity indicates significant differences in internal cell density, necrotic areas, or microvascular distribution, which may correlate statistically with heterogeneous tumor phenotypes [42]. From a statistical perspective, SHAP analysis quantifies the contribution and directional association of each radiomic feature to the prediction of recurrence, thereby identifying specific CT texture parameters as key biomarkers associated with recurrence [43, 44]. It is critical to note that while SHAP analysis successfully clarifies the mathematical directionality and relative contribution weight of individual radiomic metrics toward the model’s final prediction score, it remains a purely algorithmic attribution tool and cannot establish or validate actual biological mechanisms. The identified high-weight digital metrics serve to optimize data-driven risk stratification, and further integrative studies cross-linking these imaging phenotypes with spatial immune profiling or genomic sequencing are stringently required to decode the true underlying biological substrates. Notably, texture features derived from lymph nodes contributed significantly to the combined model, suggesting that the nodal radiomic phenotype provides unique prognostic information beyond that of the primary tumor alone [16]. This space-specific imaging phenotype underscores the macroscopic spatial heterogeneity between different tumor regions, suggesting that multi-regional radiomic features may serve as useful non-invasive surrogates statistically associated with overall tumor aggressiveness and recurrence risk.

In terms of clinical translation, this model has important reference value for “de-escalation therapy,” which is currently receiving significant attention in the field of HPV + OPC treatment. In current clinical practice, some HPV+ patients suffer from long-term complications such as xerostomia, dysphagia, and osteoradionecrosis due to overtreatment [45]. Our combined nomogram shows that the R-score plays a dominant role in risk stratification, with its contribution significantly higher than that of T-stage and N-stage. This means that for patients with extremely low radiomics scores and favorable clinical conditions, future prospective studies could explore the feasibility of shrinking target volumes or reducing radiotherapy doses to maintain survival rates while improving quality of life. Conversely, for patients with high-risk scores, even if their clinical stage is early, closer follow-up or intensified adjuvant therapy may be required. However, prospective clinical verification and safety evaluation are indispensable before formal clinical de-escalation implementation.

Despite the valuable findings of this study, several limitations remain. First, this retrospective study utilizing public datasets inevitably introduces selection bias, as evidenced by baseline imbalances in age and treatment regimens between cohorts, which might affect risk-score calibration. Future prospective studies incorporating propensity score matching or causal inference are warranted to mitigate these confounders and robustly validate model efficacy. Moreover, the data were sourced from public databases, and the lack of some clinical details may affect model performance. Second, radiomics feature extraction relies on manual target volume contouring; although consistency tests ensured the quality of contouring, subjective differences may still exist. Third, this investigation relies entirely on retrospective public repositories, meaning that critical prognostic variables—such as circulating tumor HPV DNA (ctHPV-DNA) load, molecular biomarkers, genomic sequencing data, and PET-CT metabolic parameters—were not incorporated. More importantly, although the spatial dose distribution is undeniably relevant to locoregional recurrence risk, the absence of accessible digital RT-Dose files across the multi-institutional TCIA cohorts precluded the integration of dose-volume parameters or dosiomic features, which admittedly limits the model’s direct interpretability regarding definitive radiation delivery. Nevertheless, it must be emphasized that the sub-visual anatomical textures extracted from GTVp and GTVn on Planning CT scans fundamentally capture pre-existing intrinsic biological phenotypes, such as clonal selection, non-symmetric necrosis, and hypoxic microenvironments that manifest prior to treatment execution. As a low-cost, non-invasive pre-treatment screening tool, the core utility of our model lies in isolating individuals with high baseline spatial heterogeneity who are inherently predisposed to radiation resistance. Future prospective endeavors integrating spatial dose maps, multi-parametric functional imaging, and advanced molecular oncology are strictly warranted to construct a more comprehensive multimodal prognostic framework. Future prospective multicenter studies could be conducted to integrate richer clinical, imaging, and molecular biology data to construct more comprehensive multimodal predictive models; meanwhile, AI-automated target volume contouring technology could be explored to reduce human error and improve the efficiency of clinical translation.

## Conclusion

In conclusion, the CT-based multi-regional radiomics machine learning model constructed in this study, by combining features from the primary lesion and cervical lymph nodes, significantly improves the predictive efficacy for 5-year recurrence risk in HPV + OPC patients, and has good robustness and clinical interpretability. Compared with conventional single-tumor modeling strategies, the present multi-regional model achieves improved predictive performance in this cohort and may help infer potential heterogeneity differences reflected in nodal imaging phenotypes, laying a scientific foundation for individualized recurrence risk stratification of oropharyngeal cancer and may provide preliminary evidence to inspire subsequent prospective studies to verify individualized de-escalation radiotherapy.

## Supplementary Information

Below is the link to the electronic supplementary material.

Supplementary Material 1

## Data Availability

The data that support the findings of this study are available from the corresponding author upon reasonable request.
