# Supplementary material for: Multi-regional radiomics based on planning CT and information complementarity between GTVp and GTVn: an explainable model for predicting 5-year recurrence risk in HPV-positive oropharyngeal cancer
Source: Radiat Oncol. 2026 Jul 8;21:131. doi: 10.1186/s13014-026-02889-8 (PMC13632445; doi:10.1186/s13014-026-02889-8)
Supplement: Supplementary file 1 [file 13014_2026_2889_MOESM1_ESM.docx]

Supplementary Materials for

**Multi-regional Radiomics Based on Planning CT and Information Complementarity Between GTVp and GTVn: An Explainable Model for Predicting 5-year** **Recurrence Risk in HPV-positive Oropharyngeal Cancer**

**Supplementary Tables**

**Supplementary Table 1. Baseline characteristics of the subgroup of patients with cervical lymph node metastasis**

| **Variables** | **Training set**  **n=352** | **Validation set**  **n=152** | ***P ^a^*** | **Test set**  **n=133** |
| --- | --- | --- | --- | --- |
| Age |  |  | 0.126 |  |
| ≥60 | 179(50.9%) | 80(52.6%) |  | 45(34.1%) |
| <60 | 173(49.1%) | 72(47.4%) |  | 87(65.9%) |
| Gender |  |  | 0.683 |  |
| Male | 296(84.1%) | 130(85.5%) |  | 115(87.1%) |
| Female | 56(15.9%) | 22 (14.5%) |  | 17(12.9%) |
| Smoking |  |  | 0.598 |  |
| Yes | 220(65.3%) | 103(67.8%) |  | 73(55.3%) |
| No | 122(34.7%) | 49(32.2%) |  | 59(44.7%) |
| T |  |  | 0.801 |  |
| 1-2 | 174(49.4%) | 75(49.3%) |  | 90(68.2%) |
| 3-4 | 178(50.6%) | 77(50.7%) |  | 42(31.8%) |
| N |  |  | 0.571 |  |
| 1 | 27(7.7%) | 11(7.2%) |  | 18(13.6%) |
| 2 | 294(83.5%) | 123(80.9%) |  | 111(84.1%) |
| 3 | 31(8.8%) | 18(11.8%) |  | 3(2.3%) |
| Stage |  |  | 0.596 |  |
| III | 305(86.6%) | 129(84.9%) |  | 17(12.9%) |
| IV | 47(13.4%) | 23(15.1%) |  | 115(87.1%) |
| Chemotherapy |  |  | 0.537 |  |
| Yes | 205(58.2%) | 93(61.2%) |  | 115(87.1%) |
| No | 147(41.8%) | 59(38.8%) |  | 17(12.9%) |

a: Comparison of clinical characteristics between training and validation sets.

**Supplementary Table 2. Key imaging parameters**

| Parameters | RADCURE | OPC-Radiomics | HNSCC |
| --- | --- | --- | --- |
| Slice Thickness (mm) | 2.0(2.0 – 2.5)^a^ | 2.0(2.0 – 2.5)^a^ | 1.25(1.0 – 5.0)^a^ |
| Pixel Spacing (mm) | 0.98(0.66 – 1.20)^a^ | 0.98(0.66 – 1.20)^a^ | 0.4883 |
| Number of Slices | 181(90 – 333)^a^ | 181(90 – 333)^a^ | 52 – 549 |
| Bits | 16(16 – 16)^a^ | 16(16 – 16)^a^ | 12 – 16 |
| Peak Kilovoltage (kVp) | 120(120 – 120)^a^ | 120(120 – 120)^a^ | 120(120 – 154)^a^ |
| Exposure (mAs) | 449(53 – 13742)^a^ | 449(53 – 13742)^a^ | 100 – 584 |
| Manufacturer | Toshiba  GE Medical Systems  Philips | Toshiba  GE Medical Systems  Philips | GE Medical Systems  Philips  Siemens |
| Convolution Kernel | FC04  Standard | FC04  Standard | soft  Standard |

^a^median (minimum-maximum)

**Supplementary Table 3. The radiomic features in GTV-based nomogram model**

| Name | ROI | Image type | Feature Class | Feature Name |
| --- | --- | --- | --- | --- |
| F-p 1 | GTVp | original | shape | Flatness |
| F-p 2 | GTVp | wavelet-HHH | firstorder | InterquartileRange |
| F-p 3 | GTVp | wavelet-HHH | firstorder | RobustMeanAbsoluteDeviation |
| F-p 4 | GTVp | wavelet-HLH | glszm | GrayLevelNonUniformity |
| F-p 5 | GTVp | original | glszm | SizeZoneNonUniformity |
| F-p 6 | GTVp | wavelet-HLL | glszm | SmallAreaHighGrayLevelEmphasis |
| F-p 7 | GTVp | wavelet-HHL | glrlm | LongRunHighGrayLevelEmphasis |
| F-p 8 | GTVp | wavelet-LLH | gldm | DependenceNonUniformity |
| F-p 9 | GTVp | wavelet-LLL | gldm | SmallDependenceLowGrayLevelEmphasis |
| F-p 10 | GTVp | wavelet-LLH | firstorder | Maximum |
| F-p 11 | GTVp | wavelet-HLL | glcm | Autocorrelation |
| F-p 12 | GTVp | wavelet-LLL | glcm | Idn |
| F-p 13 | GTVp | wavelet-LLH | firstorder | TotalEnergy |
| F-p 14 | GTVp | wavelet-HHL | firstorder | InterquartileRange |
| F-p 15 | GTVp | original | shape | SurfaceArea |
| F-p 16 | GTVp | wavelet-HLL | gldm | GrayLevelNonUniformity |
| F-p 17 | GTVp | wavelet-HHL | firstorder | Kurtosis |
| F-p 18 | GTVp | wavelet-LLH | glcm | ClusterProminence |

Abbreviation: F-p: Feature of GTVp.

**Supplementary Table 4. Predictive performance of the GTVp-based radiomics-score**

| Data set | Name | AUC | 95%CI | accuracy | sensitivity | specificity | PPV | NPV |
| --- | --- | --- | --- | --- | --- | --- | --- | --- |
| Training set | RF_p | 0.861 | 0.824 – 0.895 | 0.815 | 0.660 | 0.904 | 0.795 | 0.824 |
|  | MLP_p | 0.864 | 0.832 – 0.894 | 0.818 | 0.716 | 0.876 | 0.765 | 0.845 |
|  | LR_p | 0.752 | 0.701 – 0.805 | 0.697 | 0.688 | 0.703 | 0.567 | 0.799 |
|  | GNB_p | 0.749 | 0.697 – 0.805 | 0.744 | 0.567 | 0.843 | 0.672 | 0.775 |
|  | DT_p | 0.826 | 0.781 – 0.862 | 0.749 | 0.695 | 0.779 | 0.641 | 0.819 |
|  | AdaBoost_p | 0.825 | 0.778 – 0.866 | 0.795 | 0.603 | 0.904 | 0.780 | 0.801 |
|  | LightGBM_p | 0.854 | 0.817 – 0.895 | 0.774 | 0.801 | 0.759 | 0.653 | 0.871 |
|  | SVM_p | 0.841 | 0.799 – 0.880 | 0.782 | 0.794 | 0.775 | 0.667 | 0.869 |
|  | XGBoost_p | 0.865 | 0.835 – 0.891 | 0.774 | 0.830 | 0.743 | 0.646 | 0.885 |
|  | GBDT_p | 0.878 | 0.842 – 0.913 | 0.831 | 0.773 | 0.863 | 0.762 | 0.870 |
| Validation set | RF_p | 0.698 | 0.646 – 0.743 | 0.678 | 0.522 | 0.772 | 0.571 | 0.735 |
|  | MLP_p | 0.672 | 0.622 – 0.717 | 0.632 | 0.744 | 0.552 | 0.491 | 0.788 |
|  | LR_p | 0.787 | 0.708 – 0.871 | 0.754 | 0.778 | 0.714 | 0.612 | 0.847 |
|  | GNB_p | 0.831 | 0.800 – 0.860 | 0.774 | 0.717 | 0.793 | 0.668 | 0.828 |
|  | DT_p | 0.677 | 0.623 – 0.725 | 0.644 | 0.722 | 0.562 | 0.489 | 0.777 |
|  | AdaBoost_p | 0.748 | 0.654 – 0.823 | 0.735 | 0.593 | 0.786 | 0.617 | 0.769 |
|  | LightGBM_p | 0.674 | 0.635 – 0.731 | 0.636 | 0.639 | 0.636 | 0.505 | 0.752 |
|  | SVM_p | 0.826 | 0.755 – 0.883 | 0.750 | 0.944 | 0.571 | 0.561 | 0.946 |
|  | XGBoost_p | 0.772 | 0.731 – 0.810 | 0.699 | 0.672 | 0.744 | 0.604 | 0.796 |
|  | GBDT_p | 0.834 | 0.800 – 0.870 | 0.781 | 0.750 | 0.796 | 0.681 | 0.846 |
| Test set | RF_p | 0.684 | 0.612 – 0.799 | 0.657 | 0.759 | 0.561 | 0.277 | 0.913 |
|  | MLP_p | 0.743 | 0.651 – 0.811 | 0.700 | 0.704 | 0.704 | 0.345 | 0.915 |
|  | LR_p | 0.655 | 0.622 – 0.717 | 0.634 | 0.728 | 0.568 | 0.272 | 0.904 |
|  | GNB_p | 0.807 | 0.731 – 0.878 | 0.801 | 0.648 | 0.888 | 0.562 | 0.919 |
|  | DT_p | 0.657 | 0.614 – 0.702 | 0.600 | 0.767 | 0.475 | 0.244 | 0.902 |
|  | AdaBoost_p | 0.822 | 0.715 – 0.918 | 0.817 | 0.864 | 0.714 | 0.401 | 0.96 |
|  | LightGBM_p | 0.788 | 0.714 – 0.845 | 0.739 | 0.852 | 0.643 | 0.346 | 0.952 |
|  | SVM_p | 0.678 | 0.632 – 0.724 | 0.645 | 0.772 | 0.540 | 0.271 | 0.915 |
|  | XGBoost_p | 0.810 | 0.708 – 0.905 | 0.758 | 0.909 | 0.607 | 0.339 | 0.968 |
|  | GBDT_p | 0.825 | 0.780 – 0.868 | 0.774 | 0.738 | 0.796 | 0.445 | 0.932 |

Abbreviation: RF: random forest; MLP: multi-layer perceptron; LR: logistic regression; GNB: gaussian naive bayes; DT: decision tree; AdaBoost: adaptive boosting; LightGBM: light gradient boosting machine; SVM: support vector machine; XGBoost: extreme gradient boosting; GBDT: gradient boosting decision tree.

**Supplementary Table 5. Predictive performance of the GTVp-based risk-score**

| Patient sets | AUC | 95%CI | accuracy | sensitivity | specificity | PPV | NPV |
| --- | --- | --- | --- | --- | --- | --- | --- |
| Training set | 0.889 | 0.856 – 0.922 | 0.815 | 0.837 | 0.803 | 0.707 | 0.897 |
| Validation set | 0.865 | 0.810 – 0.920 | 0.813 | 0.754 | 0.848 | 0.742 | 0.856 |
| Test set | 0.847 | 0.774 – 0.919 | 0.850 | 0.655 | 0.893 | 0.576 | 0.921 |

Abbreviation: PPV: positive predictive value; NPV: negative predictive value.

**Supplementary Table 6. The radiomic features in GTVpn-based model**

| Name^a^ | ROI | Image type | Feature Class | Feature Name |
| --- | --- | --- | --- | --- |
| F-pn1 | GTVpn | wavelet-HLL | firstorder | RobustMeanAbsoluteDeviation |
| F-pn2 | GTVpn | wavelet-LLH | firstorder | Maximum |
| F-pn3 | GTVpn | original | glrlm | LongRunHighGrayLevelEmphasis |
| F-pn4 | GTVpn | wavelet-LLL | glcm | SumAverage |
| F-pn5 | GTVpn | original | glcm | DifferenceAverage |
| F-pn6 | GTVpn | wavelet-HLH | glszm | GrayLevelNonUniformity |
| F-pn7 | GTVpn | wavelet-HHL | firstorder | Minimum |
| F-pn8 | GTVpn | wavelet-LHH | firstorder | Kurtosis |
| F-pn9 | GTVpn | original | shape | MinorAxisLength |
| F-pn10 | GTVpn | wavelet-HLH | gldm | DependenceNonUniformityNormalized |
| F-pn11 | GTVpn | wavelet-HLH | glszm | LargeAreaHighGrayLevelEmphasis |
| F-pn12 | GTVpn | wavelet-LLL | firstorder | 90Percentile |
| F-pn13 | GTVpn | wavelet-HHL | firstorder | RobustMeanAbsoluteDeviation |
| F-pn14 | GTVpn | wavelet-HLL | gldm | SmallDependenceHighGrayLevelEmphasis |
| F-pn15 | GTVpn | wavelet-HHH | ngtdm | Coarseness |
| F-pn16 | GTVpn | wavelet-HLL | glszm | SmallAreaLowGrayLevelEmphasis |
| F-pn17 | GTVpn | wavelet-LHH | ngtdm | Contrast |
| F-pn18 | GTVpn | wavelet-LLL | glszm | HighGrayLevelZoneEmphasis |
| F-pn19 | GTVpn | wavelet-LHH | glcm | Autocorrelation |
| F-pn20 | GTVpn | wavelet-HLH | firstorder | Kurtosis |

Abbreviation: F-pn: Feature of GTVpn.

**Supplementary Table 7. The radiomic features in GTVp&n-based model**

| Name^a^ | ROI | Image type | Feature Class | Feature Name |
| --- | --- | --- | --- | --- |
| F-p 1 | GTVp | wavelet-LLH | firstorder | TotalEnergy |
| F-p 2 | GTVp | wavelet-LHL | gldm | LargeDependenceHighGrayLevelEmphasis |
| F-p 3 | GTVp | original | firstorder | 90Percentile |
| F-p 4 | GTVp | wavelet-LLL | ngtdm | Complexity |
| F-n 1 | GTVn | wavelet-LHH | glcm | ClusterShade |
| F-p 5 | GTVp | original | glszm | GrayLevelVariance |
| F-p 6 | GTVp | wavelet-HLL | glcm | ClusterProminence |
| F-n 2 | GTVn | original | shape | LeastAxisLength |
| F-n 3 | GTVn | wavelet-HHH | glszm | SmallAreaHighGrayLevelEmphasis |
| F-p 7 | GTVp | original | shape | Maximum2DDiameterRow |
| F-n 4 | GTVn | wavelet-HLL | glcm | DifferenceAverage |
| F-p 8 | GTVp | wavelet-HHH | glszm | LargeAreaEmphasis |
| F-n 5 | GTVn | wavelet-HHH | ngtdm | Contrast |
| F-p 9 | GTVp | original | glcm | Imc1 |
| F-p 10 | GTVp | wavelet-LHH | glszm | LowGrayLevelZoneEmphasis |
| F-p 11 | GTVp | wavelet-LHH | ngtdm | Contrast |
| F-n 6 | GTVn | wavelet-HHH | glcm | Imc2 |
| F-p 12 | GTVp | wavelet-HLL | firstorder | RobustMeanAbsoluteDeviation |
| F-n 7 | GTVn | original | glcm | JointEnergy |
| F-p 13 | GTVp | wavelet-LLL | firstorder | Minimum |

Abbreviation: F-p: Feature of GTVp; F-n: Feature of GTVn.

.**Supplementary Table 8. Predictive performance of the GTVpn-based radiomics-score**

| Name | Name | AUC | 95%CI | accuracy | sensitivity | specificity | PPV | NPV |
| --- | --- | --- | --- | --- | --- | --- | --- | --- |
| Training set | SVM_pn | 0.815 | 0.768 – 0.862 | 0.761 | 0.746 | 0.770 | 0.644 | 0.845 |
|  | RF_pn | 0.848 | 0.808 – 0.889 | 0.781 | 0.706 | 0.823 | 0.690 | 0.834 |
|  | MLP_pn | 0.856 | 0.818 – 0.895 | 0.767 | 0.865 | 0.712 | 0.626 | 0.904 |
|  | LR_pn | 0.857 | 0.817 – 0.896 | 0.761 | 0.889 | 0.690 | 0.615 | 0.918 |
|  | GNB_pn | 0.770 | 0.721 – 0.820 | 0.699 | 0.760 | 0.664 | 0.558 | 0.833 |
|  | LightGBM_pn | 0.874 | 0.836 – 0.912 | 0.818 | 0.770 | 0.845 | 0.735 | 0.868 |
|  | GBDT_pn | 0.873 | 0.836 – 0.911 | 0.807 | 0.801 | 0.810 | 0.701 | 0.880 |
|  | AdaBoost_pn | 0.848 | 0.809 – 0.887 | 0.747 | 0.810 | 0.712 | 0.611 | 0.870 |
|  | DT_pn | 0.802 | 0.757 – 0.848 | 0.741 | 0.754 | 0.735 | 0.612 | 0.843 |
|  | XGBoost_pn | 0.887 | 0.852 – 0.923 | 0.795 | 0.881 | 0.748 | 0.661 | 0.918 |
| Validation set | SVM_pn | 0.775 | 0.694 – 0.845 | 0.730 | 0.852 | 0.592 | 0.535 | 0.879 |
|  | RF_pn | 0.693 | 0.599 – 0.764 | 0.685 | 0.759 | 0.643 | 0.539 | 0.829 |
|  | MLP_pn | 0.689 | 0.641 – 0.736 | 0.634 | 0.460 | 0.867 | 0.656 | 0.744 |
|  | LR_pn | 0.681 | 0.612 – 0.733 | 0.663 | 0.492 | 0.788 | 0.561 | 0.738 |
|  | GNB_pn | 0.830 | 0.791 – 0.869 | 0.746 | 0.817 | 0.694 | 0.595 | 0.873 |
|  | LightGBM_pn | 0.815 | 0.768 – 0.858 | 0.764 | 0.746 | 0.770 | 0.641 | 0.846 |
|  | GBDT_pn | 0.740 | 0.655 – 0.823 | 0.738 | 0.630 | 0.765 | 0.596 | 0.79 |
|  | AdaBoost_pn | 0.829 | 0.798 – 0.860 | 0.771 | 0.635 | 0.861 | 0.716 | 0.811 |
|  | DT_pn | 0.678 | 0.619 – 0.720 | 0.638 | 0.711 | 0.580 | 0.483 | 0.785 |
|  | XGBoost_pn | 0.838 | 0.801 – 0.874 | 0.787 | 0.733 | 0.796 | 0.664 | 0.844 |
| Test set | SVM_pn | 0.708 | 0.624 – 0.770 | 0.716 | 0.796 | 0.574 | 0.325 | 0.916 |
|  | RF_pn | 0.677 | 0.618 – 0.726 | 0.643 | 0.706 | 0.580 | 0.302 | 0.885 |
|  | MLP_pn | 0.714 | 0.669 – 0.754 | 0.667 | 0.605 | 0.744 | 0.378 | 0.88 |
|  | LR_pn | 0.688 | 0.590 – 0.765 | 0.679 | 0.741 | 0.592 | 0.318 | 0.899 |
|  | GNB_pn | 0.693 | 0.646 – 0.753 | 0.672 | 0.683 | 0.648 | 0.333 | 0.888 |
|  | LightGBM_pn | 0.824 | 0.785 – 0.857 | 0.770 | 0.739 | 0.781 | 0.465 | 0.921 |
|  | GBDT_pn | 0.809 | 0.766 – 0.848 | 0.751 | 0.772 | 0.772 | 0.465 | 0.929 |
|  | AdaBoost_pn | 0.728 | 0.590 – 0.828 | 0.735 | 0.636 | 0.804 | 0.455 | 0.896 |
|  | DT_pn | 0.700 | 0.616 – 0.787 | 0.690 | 0.741 | 0.643 | 0.348 | 0.906 |
|  | XGBoost_pn | 0.825 | 0.742 – 0.884 | 0.817 | 0.593 | 0.949 | 0.749 | 0.901 |

Abbreviation: RF: random forest; MLP: multi-layer perceptron; LR: logistic regression; GNB: gaussian naive bayes; DT: decision tree; AdaBoost: adaptive boosting; LightGBM: light gradient boosting machine; SVM: support vector machine; XGBoost: extreme gradient boosting; GBDT: gradient boosting decision tree.

**Supplementary Table 9. Predictive performance of the GTVp&n-based radiomics-score**

| Name | Name | AUC | 95%CI | accuracy | sensitivity | specificity | PPV | NPV |
| --- | --- | --- | --- | --- | --- | --- | --- | --- |
| Training set | DT_p&n | 0.813 | 0.768 – 0.858 | 0.744 | 0.865 | 0.677 | 0.600 | 0.900 |
|  | SVM_p&n | 0.862 | 0.823 – 0.902 | 0.801 | 0.738 | 0.836 | 0.715 | 0.851 |
|  | RF_p&n | 0.871 | 0.832 – 0.909 | 0.835 | 0.659 | 0.934 | 0.847 | 0.831 |
|  | MLP_p&n | 0.867 | 0.828 – 0.905 | 0.801 | 0.778 | 0.814 | 0.700 | 0.868 |
|  | LR_p&n | 0.846 | 0.805 – 0.888 | 0.739 | 0.849 | 0.677 | 0.594 | 0.890 |
|  | GNB_p&n | 0.825 | 0.779 – 0.870 | 0.776 | 0.738 | 0.796 | 0.669 | 0.845 |
|  | AdaBoost_p&n | 0.871 | 0.832 – 0.910 | 0.784 | 0.817 | 0.765 | 0.660 | 0.883 |
|  | GBDT_p&n | 0.895 | 0.861 – 0.929 | 0.835 | 0.801 | 0.854 | 0.754 | 0.885 |
|  | LightGBM_p&n | 0.880 | 0.845 – 0.916 | 0.795 | 0.841 | 0.700 | 0.671 | 0.897 |
|  | XGBoost_p&n | 0.907 | 0.877 – 0.938 | 0.844 | 0.762 | 0.889 | 0.793 | 0.870 |
| Validation set | DT_p&n | 0.674 | 0.592 – 0.752 | 0.650 | 0.759 | 0.582 | 0.5 | 0.814 |
|  | SVM_p&n | 0.705 | 0.627 – 0.790 | 0.685 | 0.574 | 0.765 | 0.574 | 0.765 |
|  | RF_p&n | 0.762 | 0.721 – 0.803 | 0.718 | 0.733 | 0.713 | 0.585 | 0.829 |
|  | MLP_p&n | 0.837 | 0.806 – 0.869 | 0.769 | 0.806 | 0.719 | 0.612 | 0.871 |
|  | LR_p&n | 0.671 | 0.611 – 0.733 | 0.638 | 0.738 | 0.544 | 0.471 | 0.79 |
|  | GNB_p&n | 0.846 | 0.813 – 0.874 | 0.756 | 0.917 | 0.636 | 0.581 | 0.933 |
|  | AdaBoost_p&n | 0.770 | 0.727 – 0.810 | 0.711 | 0.762 | 0.664 | 0.555 | 0.835 |
|  | GBDT_p&n | 0.851 | 0.819 – 0.882 | 0.768 | 0.850 | 0.707 | 0.615 | 0.895 |
|  | LightGBM_p&n | 0.848 | 0.810 – 0.881 | 0.788 | 0.706 | 0.823 | 0.687 | 0.836 |
|  | XGBoost_p&n | 0.852 | 0.815 – 0.879 | 0.822 | 0.656 | 0.920 | 0.819 | 0.829 |
| Test set | DT_p&n | 0.655 | 0.597 – 0.715 | 0.640 | 0.548 | 0.717 | 0.332 | 0.861 |
|  | SVM_p&n | 0.716 | 0.635 – 0.792 | 0.694 | 0.759 | 0.643 | 0.353 | 0.912 |
|  | RF_p&n | 0.703 | 0.661 – 0.751 | 0.649 | 0.469 | 0.865 | 0.472 | 0.864 |
|  | MLP_p&n | 0.767 | 0.732 – 0.802 | 0.710 | 0.817 | 0.636 | 0.366 | 0.931 |
|  | LR_p&n | 0.693 | 0.615 – 0.764 | 0.690 | 0.611 | 0.735 | 0.372 | 0.88 |
|  | GNB_p&n | 0.827 | 0.791 – 0.860 | 0.762 | 0.772 | 0.765 | 0.458 | 0.929 |
|  | AdaBoost_p&n | 0.802 | 0.754 – 0.846 | 0.742 | 0.754 | 0.735 | 0.423 | 0.921 |
|  | GBDT_p&n | 0.813 | 0.771 – 0.851 | 0.743 | 0.865 | 0.677 | 0.408 | 0.951 |
|  | LightGBM_p&n | 0.825 | 0.759 – 0.886 | 0.794 | 0.722 | 0.837 | 0.532 | 0.921 |
|  | XGBoost_p&n | 0.835 | 0.797 – 0.868 | 0.760 | 0.867 | 0.679 | 0.41 | 0.952 |

Abbreviation: RF: random forest; MLP: multi-layer perceptron; LR: logistic regression; GNB: gaussian naive bayes; DT: decision tree; AdaBoost: adaptive boosting; LightGBM: light gradient boosting machine; SVM: support vector machine; XGBoost: extreme gradient boosting; GBDT: gradient boosting decision tree.

**Supplementary Table 10. Predictive performance of the GTVp&n-based risk-score**

| Patient sets | AUC | 95%CI | accuracy | sensitivity | specificity | PPV | NPV |
| --- | --- | --- | --- | --- | --- | --- | --- |
| Training set | 0.934 | 0.908 – 0.959 | 0.855 | 0.913 | 0.823 | 0.742 | 0.944 |
| Validation set | 0.920 | 0.877 – 0.963 | 0.842 | 0.889 | 0.816 | 0.727 | 0.930 |
| Test set | 0.908 | 0.842 – 0.973 | 0.841 | 0.852 | 0.838 | 0.575 | 0.957 |

Abbreviation: PPV: positive predictive value; NPV: negative predictive value.

**Supplementary Figures**

**Supplementary Figure 1. Radiomics feature selection and model performance comparison based on GTVpn and GTVp&n.** (A). LASSO logistic regression feature selection curves for the GTVpn model: the left plot shows the trend of binomial deviance with the logarithm of the penalty parameter (Log Lambda), while the right plot shows the trajectories of coefficients for 20 selected features relative to Log Lambda. (B). Feature importance bar chart for the GTVp&n model, showing the distribution of weight coefficients for 20 features fused from the GTVp and GTVn regions. (C). Confusion matrices for the GTVpn model. (D). ROC curve comparison for the GTVpn model. (E). ROC curve comparison for the GTVp&n model. (F). SHAP summary plot for the GTVp&n model.
